# Supplementary material for: Reconstructing Native American Migrations from Whole-Genome and Whole-Exome Data
Source: PLoS Genet. 2013 Dec 26;9(12):e1004023. doi: 10.1371/journal.pgen.1004023 (PMC3873240; doi:10.1371/journal.pgen.1004023)
Supplement: Text S1 — Supplementary methods include additional description of statistical and filtering methods used in this article. (PDF) [file pgen.1004023.s008.pdf]

Text S1: Reconstructing Native American Migrations from Whole-genome and Whole-exome Data

IBD and local ancestry blocks

For all individuals presented here, we retrieved phased local ancestry inference results generated using RFMIX as part of the 1000 Genomes project [1]. The accuracy of the RFMix algorithm has been tested under a wide range of scenarios in [2], and was found to be robust to the likely amount of divergence between the actual and proxy ancestral populations used here.

IBD tracts across individuals were obtained using GERMLINE [3] with the 'phased' (-h_extend) option and the 1000 Genomes recombination map. Because this method tends to overcall IBD around centromeres, we discarded regions with over 100 inferred IBD segments, and only IBD segments spanning 3Mb on each side of these regions were considered as spanning the regions. Finally, individuals NA19657, NA19661, NA19660, and NA19675 were not used in the IBD analysis due to known or inferred relatedness with other samples.

Because the ADMIXTURE and AS-PCA analyses used comparisons to a Native American panel [4] different from the panel used to infer local ancestry in the 1000 Genomes [5], we re-inferred local ancestry using the panel from [4] for the purpose of these analyses.

ADMIXTURE and AS-PCA

We developed software tools to perform principal-component analysis within fractions of continental ancestry Moreno-Estrada et al, PLoS Genetics, in Press. For all ADMIXTURE and PCA analyses, we used a combination of data from the 1000 Genomes and 493 Native American samples from [4]. These Native American samples are drawn from 52 populations specified in supplementary Table 1 of [4]. We had access to genotypes at 364,470 SNPs from the Illumina 650K platform. To perform the analysis jointly with 1000 Genomes OMNI data, we restricted the analysis to the 207,430 SNPs in the intersection of the two genotyping platforms. ADMIXTURE was run for K=2-14, and plotted following a North-South order within the NAT groups (Figure S6).

Calculating the site-frequency spectrum

The original data in vcf format was downloaded from the 1000 Genomes server: `ftp://ftp-trace.ncbi.nih.gov/1000genomes/ftp/release/20110521`. Frequency-based demographic analyses were restricted to parts of the genome that were within the consensus exome target and that passed the strictest callability mask from the 1000 Genomes project. One individual from each pair with cryptic relatedness were removed. This resulted in discarding 7 MXL individuals, NA19661, NA19675, NA19660, NA19657, NA19664, NA19672, and NA19726.

Diploid local ancestry assignments were obtained from the 1000 Genomes server: `ftp://ftp-trace.ncbi.nih.gov/1000genomes/ftp/phase1/analysis_results/ancestry_deconvolution`, for the CLM, MXL, and PUR. Diploid assignments could be any pair drawn from African, European, or Native American ancestries, or Unassigned.

Reference and nonreference allele frequencies were tabulated for each population and ancestry, and SNPs were

annotated according to Gencode version 13. Alignment with chimp was performed using LIFTOVER, the PanTro3 chimpanzee genome, and the corresponding alignment files from UCSC website.

To estimate the expected number of synonymous sites in the callable region, we used the Hwang-Green mutational model [6]. The Hwang-Green model provides 4^4 mutational rates for sites with different immediate neighboring bases. Each site was allowed to mutate proportionally to this context dependent rate, and the overall counts were normalized to obtain an average of one annotation per site. We obtained an expected total of $5,030,734$ synonymous sites.

Estimating theta and the mutation rate

When performing demographic inference using ∂a∂i, we calculated the expected frequency spectrum assuming a unit-size initial population. All the parameters in the demographic model are scaled by the actual size of the initial population size, which can be obtained as the ratio of the number of segregating sites observed in the data, to that expected under the unit-population-size model. The first one is easily computed, but the second one requires an estimate the number of sites sequenced. From the total estimated number of $5,030,734$ synonymous sites, we need to remove sites that would not have passed downstream filters. Because we focused on sites that had minimal amounts of Native American ancestry, we discarded a different proportion of sites for each pairwise SFS– the fraction of sites that passed all filters was 0.895, 0.737, and 0.693 in the CLM-MXL, PUR-MXL, PUR-CLM pairwise spectra. We obtain a slightly different theta value for each pairwise spectrum; we used the mean of these three values in the rest of the analysis. Finally, we need to fix either the human mutation rate, or the time of an event in our demographic model. We used 16 kya as a reference point for the population recovery into the Americas, and inferred the mutation rate based on this value.

Confidence intervals for demographic parameters through the SFS

We estimated confidence intervals induced by the finiteness of the genome by bootstrapping over contiguous genomic loci. The genome was divided at every segment of at least 100kb with no sequence data. The resulting 4493 'loci' were then sampled with replacement 100 times, and the complete inference pipeline was run on the resulting target region: estimation of the singleton error rates, inference of genetic parameters, and conversion to physical units by estimating the effective synonymous sequenced length. The resulting confidence intervals are shown on Table 1.

Significance of allele frequency difference

We calculated the 95% confidence interval width w for the allele frequency in the Native American components at each position in each population panel. To estimate the significance of the allele frequency difference among two groups, we fitted a Binomial distribution to the frequency and confidence interval observed in each population, and performed a parametric bootstrap with 3×10^7 iterations, counting the number of occurrences where the ordering is inverted. Using a Bonferroni correction with 29,354 tests, a bootstrap with 0 inverted samples would yield $p < 0.001$.

Simulating recent bottlenecks

The piecewise-constant model of Native American population sizes considered in the main text does not allow for recent fluctuations in the Native population sizes. However, many Native American populations suffered drastic reductions in population sizes after the arrival of Europeans. We expect that the piecewise constant population sizes are effective sizes that average out the real, fluctuating population size. If we wish to interpret these effective population sizes in terms of pre-Colombian population sizes, we must account for the possible effect of recent bottlenecks in these inference results. We also wanted to study the effect of the bottlenecks on other model parameters, such as the split times.

We focused out attention on the PUR population because 1) history suggests a particularly drastic bottleneck, 2) historical data about this bottleneck is more detailed than for other populations, and 3) it has the lowest effective population size, suggesting that the recent, strict bottleneck might explain the difference in inferred population sizes. In Puerto Rico, the Native population prior to the arrival of Europeans was estimated at slightly above 110,000 individuals [7]. After contact, the population decreased rapidly. It is difficult to estimate the extent of this bottleneck due to incomplete and biased post-contact census data. To get an order-of-magnitude estimate, we note that the total census population of San Juan in 1673 was 1523. If we suppose that San Juan represented 10% of the Island population (the figure from 1765, when such data becomes available), and that the Native American ancestry proportion in the population by then was in equal proportion to what it is today (13%), we have an aggregated Native American population (aNA, see next section) of about 2000 Native Americans. Assuming that the effective population size bottleneck is proportional to this census population size bottleneck, we may consider a 1.5% bottleneck lasting from 1500 to 1750. By 1765, the island population is 45,000 (aNA=$5,850$) and rapidly expands thereafter, and we consider that the bottleneck had ended by that point.

We first attempted to estimate the size of the bottleneck from the data by re-estimating all parameters and letting the depth of the bottleneck vary. We did not find this to significantly increase the model likelihood, confirming that we do not have the power to differentiate between the no-bottleneck and the bottleneck case. To estimate the possible effect of the bottleneck on parameter inference and on the pre-Columbian population size estimates, we imposed the 1.5% bottleneck and optimized all other parameters (including the population size in the PUR branch). As expected, the inferred population size in the PUR population was increased, by a factor of 3.9. It remained lower than the CLM estimated population size (without bottleneck). The maximum change in any other parameter was a 10% increase in the PUR/CLM split time.

To further test the robustness of split time inference to the presence of bottlenecks, we introduced a second bottleneck of identical duration and depth in the CLM population. We found a similar 4-fold increase of CLM and PUR pre-bottleneck population sizes, relative to the no-bottleneck models. Both population size estimates remained well below the MXL estimate. We found modest parameter changes for other parameters: a 20% increase in the MXL population size estimate, and increases of 9.9% in the MXL split time and 8.4% in the CLM/PUR split time.

Finally, we can obtain an order-of-magnitude estimate of the change in effective population size due to a bottleneck using the harmonic mean formula for drift: if the bottleneck lasts a fraction ρ of the current time period, and the population reduces to size αN, we get $\hat{N}_e = \frac{N}{\rho/\alpha+(1-\rho)}$. In the current model, $\rho = 0.019$, and $\alpha = 0.015$, so that $\hat{N}_e = 2.2N$. However, this estimate does not account for new mutations occurring during the time interval. Because such mutations represent a considerable proportion of mutations in our model, the harmonic mean estimates don't provide accurate results, but may help in quickly assessing the effects of different bottleneck models.

Aggregated effective population size

We wish to model the allele frequency within the Native American component of admixed populations as if it was evolving under a randomly mating population of a given effective size. The natural choice would be to use an effective population size equal to the average number of Native American haplotypes in the population. We call this the aggregate Native American population size, since it represents an effective size across of Native American ancestry aggregated over all individuals. In this section, we show that this is reasonable.

Given that we have N Native American alleles at generation t, pN of which carry allele a and $(1-p)N$ of which carry allele A, the variance in p', the proportion of allele a in the next generation, can be expressed using the law of total variance $\mathrm{Var}(p') = E'_N(\mathrm{Var}(p'|N')) + \mathrm{Var}'_N(E(p'|N'))$, where N' is the number of Native American alleles at the next generation. Because $E(p'|N') = p$ is independent of N', the second term is zero. The first term is

$$E'_N(\mathrm{Var}(p'|N')) = E'_N(p(1-p)/N') = p(1-p)E'_N(1/N').$$

The latter term is infinite because of the ever so slight probability that no Native haplotype remains, which would lead to an indeterminate value for p. Because we do not calculate allele frequencies in such cases, we find that to a very good approximation (for $N > 100$) $E'_N(1/N') \simeq 1/N$. Thus we find $\mathrm{Var}(p') = p(1-p)/N$. As could be expected, the variation in allele frequency behaves roughly as it would if we tracked allele frequencies in a total population size of size N, the expected number of Native American alleles per site. Because the proportion of Native American ancestry can vary from site to site due to drift, we have a drift term for $\mathrm{Var}(p')$. In other words, there is drift on the drift parameter. However, given the short time since admixture in the present study, we will neglect such second-order drift.

Significance of IBD vs Ancestry results

We wish to determine whether long IBD tracts have a higher density of ancestry switch-point, given that they are likely to have more recent TMRCAs. To do so, we first discarded 2cM from each edge of IBD tracts, because the IBD boundaries are likely to also be ancestry switch-points, and small errors in the estimated positions of switch-points can inflate the number of inferred switches within the tracts. We calculated the number and density of switch-points in the interior region. We then sorted the IBD segments according to length, grouped them in blocks of n IBD segments, and computed the mean length and switch-point density. This grouping allows us to calculate the uncertainty in each block via the bootstrap–we chose $n = 40$ in the MXL and $n = 200$ in the other two populations. Much smaller block sizes result in risking some bins containing only IBD segments without switch points, making the bootstrap analysis meaningless. Because MXL has only 307 IBD segments of sufficient length, smaller block sizes had to be used.

Once the bin-specific variances have been estimated, we computed a linear regression on the mean values for each bin using the original sample and 1000 bootstrapped samples. The reported p-values are the fraction of bootstrap instances that have non-positive correlation.

Results appeared robust to increasing n in populations where a signal was observed, PUR ($n = 400, p < 0.001$) and MXL ($n = 153$, $p = 0.012$). CLM remained insignificant.

Confidence intervals and goodness-of-fit in TRACTS

Reference [8] described a likelihood-ratio test to compare different migration models, but not a goodness-of-fit test. In our model assumptions, the number of tracts t_i in each length bin is a Poisson variable with mean e_i given by the model expectation. Thus we can calculate Pearson's χ^2 statistic

$$X = \sum_{i=1}^{B} \frac{(t_i - e_i)^2}{e_i}, \tag{1}$$

where B is the total number of bins after bins with less than 10 expected counts per bin in each population have been pooled. We model this as a χ^2_{B-1-n} distribution, with n the number of fitted parameters.

As a starting point in CLM and PUR, we considered a model in which Europeans and Native Americans first form a panmictic admixed population, which subsequently receives migrants from an African source population. The timing and magnitude of each migration is chosen to maximize the model likelihood. This model has four free parameters: timing and ancestry proportions at the first generation, and timing and magnitude of the African migration.

In Puerto Ricans, we found that the model is significantly improved upon if a second pulse of migration is allowed for both European and African ancestry: adding a pulse of African migrants at the population onset improves the log-likelihood by 17 units, and the second European migration epoch further improves the log-likelihood by 13 units. This more than justifies the three extra parameters according to the Bayesian information criterion with 150 data points. The p-value of a χ^2 goodness-of-fit test for the final optimized model, displayed in Figure 3a, was 0.50, indicating that this model accurately describes the data.

In the CLM, incorporating additional recent migration from both Europeans and Native Americans improves the fit: the additional European pulse adds 53 log-likelihood units, whereas the Native American pulse adds 14, again justifying the most complex model using the Bayesian information criterion. The final χ^2 p-value is 0.47. The best-fitting model is displayed in Figure 3b.

In MXL, the χ^2 goodness-of-fit p-value was 0.017 for the model considered in [9], indicating that it may be possible to marginally improve upon this model.

To estimate confidence intervals on specific parameters of the migration model, we performed a bootstrap analysis by sampling *individuals* with replacement. Thus these confidence intervals are robust to population structure. The optimal parameters and confidence intervals are provided in Figure S1

Timing recombinations and TMRCA using IBD and ancestry patterns

Formally, the likelihood function for the demographic model θ and TMRCA t, given an IBD length of ℓ and an ancestry pattern a is

$$P(a, \ell \| t, \Theta) = P(a \| \ell, t, \Theta) P(\ell \| t, \Theta).$$

The first expression can be obtained using a Markov model developed in [8], the second using the IBD models developed in [10]. Because the amount of IBD increases rapidly with sample size, we expect such approaches to be particularly suited for large genotyping cohorts.

Table S1. Parameters and confidence intervals for migration parameters inferred using TRACTS. T's are time in generations and p's are proportion of migrants per generation. p's with numbered labels correspond to punctual migrations at the correspondingly numbered time. Because the models allow only migrations at integer times, migrations occurring at non-integer times were distributed over neighboring integer migrations with higher weight given to the more nearby value. p's with no numbered labels (in MXL) correspond to continuous migrations. Migration proportions at T_0 are constrained to sum to one. In MXL, an additional constraint was $p_{Eu0}/p_{Na0} = p_{Eu}/p_{Na}$.

	PUR			CLM			MXL	
param.	estim.	95% CI	param.	estim.	95% CI	param.	estim.	95% CI
T_0	14.9	$(14.2, 15.9)$	T_0	13.0	$(12.5, 13.9)$	T_0	15.1	$(13.7, 17.1)$
p_{Af0}	0.103	$(0.085, 0.132)$	p_{Eu0}	0.690	$(0.659, 0.712)$	p_{Af0}	0.109	$(0.078, 0.160)$
p_{Eu0}	0.702	$(0.606, 0.735)$	p_{Na0}	0.310	$(0.288, 0.341)$	p_{Eu0}	0.453	$(0.405, 0.496)$
p_{Na0}	0.195	$(0.169, 0.261)$	T_1	9.6	$(8.7, 10.8)$	p_{Na0}	0.438	$(0.385, 0.480)$
T_1	6.8	$(5.9, 8.8)$	p_{Af1}	0.077	$(0.056, 0.107)$	p_{Eu}	0.037	$(0.028, 0.046)$
p_{Af1}	0.042	$(0.017, 0.066)$	T_2	4.8	$(4.0, 6.0)$	p_{Na}	0.036	$(0.027, 0.045)$
p_{Eu1}	0.268	$(0.150, 0.445)$	p_{Eu2}	0.141	$(0.098, 0.213)$			
			p_{Na2}	0.013	$(0.003, 0.035)$			

References

1. 1000 Genomes Project Consortium (2012) An integrated map of genetic variation from 1,092 human genomes. Nature 491: 56–65.

2. Maples BK, Gravel S, Kenny EE, Bustamante CD (2013) RFMix: A Discriminative Modeling Approach for Rapid and Robust Local-Ancestry Inference. Am J Hum Genet 93: 278–288.

3. Gusev A, Lowe JK, Stoffel M, Daly MJ, Altshuler D, et al. (2008) Whole population, genome-wide mapping of hidden relatedness. Genome Res 19: 318–326.

4. Reich D, Patterson N, Campbell D, Tandon A, Mazieres S, et al. (2012) Reconstructing Native American population history. Nature 488: 370–374.

5. Mao X, Bigham AW, Mei R, Gutierrez G, Weiss KM, et al. (2007) A genomewide admixture mapping panel for Hispanic/Latino populations. Am J Hum Genet 80: 1171–1178.

6. Hwang DG, Green P (2004) Bayesian Markov chain Monte Carlo sequence analysis reveals varying neutral substitution patterns in mammalian evolution. Proc Natl Acad Sci USA 101: 13994–14001.

7. Moscoso F (2008) Caciques, aldeas y población taína de Boriquén (Puerto Rico), 1492-1582. Academia puertorriquena de la historia.

8. Gravel S (2012) Population genetics models of local ancestry. Genetics 191: 607–619.

9. Kidd JM, Gravel S, Byrnes J, Moreno-Estrada A, Musharoff S, et al. (2012) Population genetic inference from personal genome data: impact of ancestry and admixture on human genomic variation. Am J Hum Genet 91: 660–671.

10. Palamara PF, Lencz T, Darvasi A, Pe'er I (2012) Length distributions of identity by descent reveal fine-scale demographic history. Am J Hum Genet 91: 809–822.
